# Recent Resolution by the Royal College of Physicians

**Published:** 1882-02

**Authors:** 


### RECENT RESOLUTION BY THE ROYAL COLLEGE OF PHYSICIANS.

The Royal College of Physicians of England has recently adopted the following resolution:

"While the College has no desire to fetter the opinion of its members in reference to any theories they may see fit to adopt in connection with the practice of medicine, it nevertheless considers it desirable to express its opinion that the assumption or acceptance, by the members of the profession, of designations implying the adoption of special modes of treatment, is opposed to those principles of the freedom and dignity of the profession which should govern the relations of its members to each other and to the public. The College therefore expects that all its fellows, members, and licentiates will uphold these priuciples by discountenancing those who trade upon such designations."

If this resolution means anything, it means that a man may believe in Homœopathy and practise it to his heart's content, but he may not call himself a homœopath; he may believe that there is nothing like cold water, and he may dose his patients with it internally, externally, and eternally, but he must not call himself a hydropath; he may believe electricity to be the most potent healing agent in existence, and may confine his practice to its administration, but he must not call himself an electropath; he may believe that all diseases are more quickly cured by venesection, but he must not call himself a phlebotomist. In other words, he may think as he pleases, practice as he pleases, but he must not assume a party designation.

What could have induced the Royal College to adopt a resolution so contrary to all its precedents for the past thirty years,it is not easy to say. Is this concession to liberty of opinion and practice due to the influence of Ringer, whose writings contain not a little clandestine Homœopathy. Is it due to the influence of Phillips, who for fifteen or twenty years was a professed homœopath, and whose writings clearly indicate his special training in homœopathic therapeutics? These questions we cannot answer. At a distance of three thousand miles it would be difficult—in fact, impossible—to accurately judge of the influences and motives that induced the Royal College to unanimously adopt the foregoing resolution, which, on debate, was supported by Dr. Priestly, Dr. Wilks, Dr. Beale, Dr. Wilson Fox, Sir W. Gull, Sir W. Jenner, and other noted medical men of the British metropolis. Verily "*tempora mutantur*," etc.

This resolution, guarnteeing liberty of opinion and practice, may be taken as a direct invitation to homœopaths and other dissenters and sectarians to abandon their special designations, and to place themselves in relationship with the College. It remains to be seen whether these gentlemen will accept this invitation—whether they will dare to accept it; whether they will dare to appear before the public simply as physicians relying for success on their individual merit and practical application of what they claim to be superior methods of treatment. The Royal College of Physicians of England has offered the invitation and the challenge; it is yet to be seen whether the sectarians of Great Britain will accept them.—*Med. Record* (Jan. 21, 1882).

[Allopathic "resolutions" concerning Homœopathy are about as frequent and as impotent as the New Year resolutions of a toper. Yet, we like to see our dear brethren resolve, for it shows that Homœopathy is stirring them up, as pure Homœopathy can always do.]

